# Supplementary material for: Enhanced Wound Healing Potential of Spirulina platensis Nanophytosomes: Metabolomic Profiling, Molecular Networking, and Modulation of HMGB-1 in an Excisional Wound Rat Model
Source: Mar Drugs. 2023 Feb 24;21(3):149. doi: 10.3390/md21030149 (PMC10051127; doi:10.3390/md21030149)
Supplement: Supplementary file 1 [file marinedrugs-21-00149-s001.zip › marinedrugs-2214324-supplementary.pdf]

Supplementary Material

A) Characterization of SPNP-Gel

1. Methodology

1.1. Organoleptic Properties and pH Measurement

The appearance, color, homogeneity, and clarity of the gel were visually evaluated under light against white and black backgrounds. The pH value of the appropriately diluted prepared gel (1:10) was measured in triplicates using a calibrated pH meter (InolabpH720, WTW, Germany) at room temperature.

1.2. Drug Content

The drug content of the gel was determined by accurately weighing a gel sample and dissolving it in 100 ml of methyl alcohol. The resultant solution was filtered through 0.45 µm membrane filter before being tested spectrophotometrically at 273 nm [1].

$$\text{drug content\%} = \frac{actual\ amount\ (1\ gm\ gel)}{theoretical\ amount\ (1gm\ gel)} \text{ X } 100$$

1.3. Rheology Measurement

Rheological measurements were performed using a rotational cone and plate Brookfield viscometer (Brookfield programmable DVII + Model pro II type, USA) with a cone spindle #52 at a temperature of 25 ± 1 ºC [2]. A small amount of the measured gel about (1 g) was added to the plates, leaving the cone at a temperature of 25 ± 1 ºC, and the shear rate was increased gradually in a suitable range to give torque values between 0.1 to 240 rpm, with 30 sec between every 2 successive points. Gel's flow characteristics (η_{min}, η_{max}, Farrow's constant, and hysteresis loop area) were determined by plotting the shear rate against the shear stress [3]. To predict the rheological behavior of the formulated gel, the obtained data were fitted to Farrow's equation [4]

$$\text{Farrow's equation: } \text{Log } G = N \text{ Log } F - \text{Log } \eta$$

where:

G is the shear rate (sec^{-1}), F is shear Stress (Pa), N is Farrow's constant, and η: viscosity (mPa·s). (N < 1) indicate for shear-thickening systems, while for Newtonian systems, N approaches 1, whereas N exceeds 1 in the case of shear-thinning systems.

1.4. Spreadability

The gel's spreadability was assessed by using two glass slides. The gel (0.5 g) was placed over one of them and the other one was placed on top of it. A fixed weight was put over the upper slide for approximately 5 minutes, allowing the gel to be pushed uniformly to create a thin layer with no further spreading expected. The gel's travel distance was calculated [5].

2. Results and Discussion

2.1. Organoleptic Properties and pH Measurement

The visual inspection of the systems revealed the development of translucent and homogenous gel with no signs of separation or precipitation. The pH was 6.95 which lies within the normal pH range of the skin. It could be assumed that the system is acceptable and would not produce any skin irritation.

2.2. Drug Content

The drug content was 98.1% ± 0.15 which is within the limits of the pharmacopeial range [6], suggesting the homogeneity of the drug in the gel matrix.

2.3. Rheology Measurement

SPNP-Gel showed non-Newtonian pseudoplastic (shear thinning) flow (Fig. S1), this is confirmed by the flow index (n) value of 0.56, and Farrow's constant (N) of 2.67 [3]. The minimum and the maximum viscosity values of the gel were 714.4 and 3493 respectively. Thixotropy is measured quantitatively by the area of the hysteresis loop; the larger the area, the greater the increase in thixotropy [7]. The hysteresis loop area was 2.04 which showed good thixotropic behavior (Fig. S2).

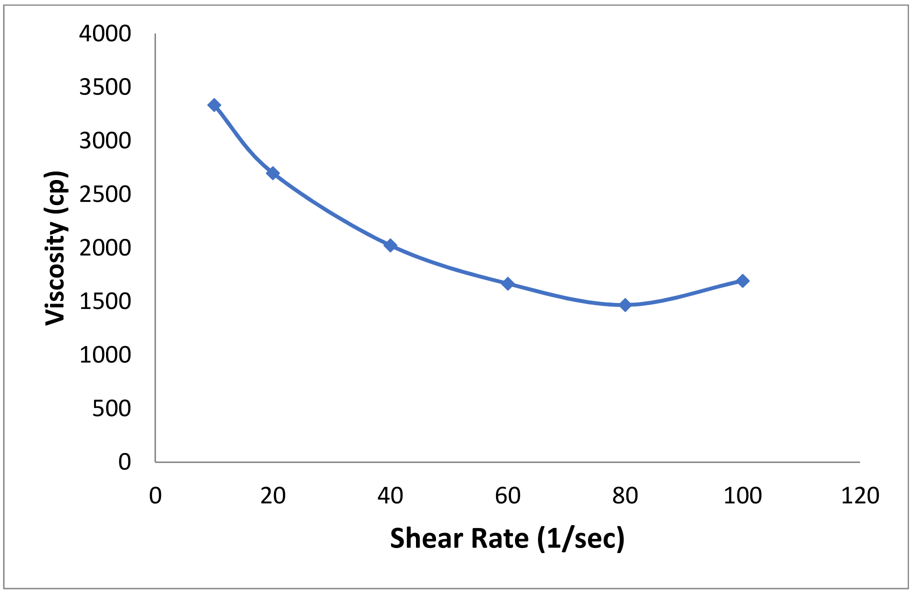

Figure S1 The Rheological behavior of SPNP-Gel

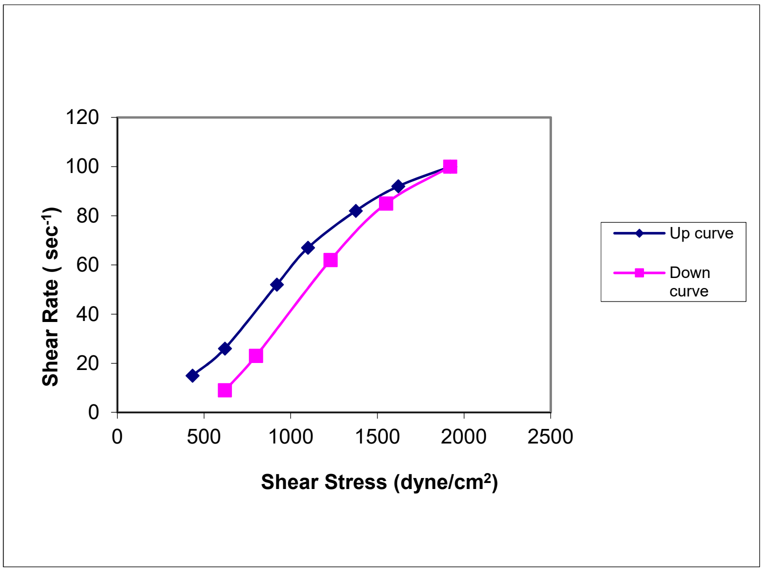

Figure S2 Thixotropic behavior of SPNP-Gel

2.3. Spreadability

The spreadability of SPNP-gel was found to be 5.8± 0.3 cm indicating good spreadability [5].

<u>B) Release Profile of Formulated Nanophytosomal Dispersions</u>

The release profiles of the formulated nanophtosomal dispersions are shown in Fig. S3. It could be noticed that the nanophytosomal dispersions showed similar drug release for up to 4 h, after

which F4 showed significantly greater drug release than the other formulae. This finding is discussed in detail in the main text.

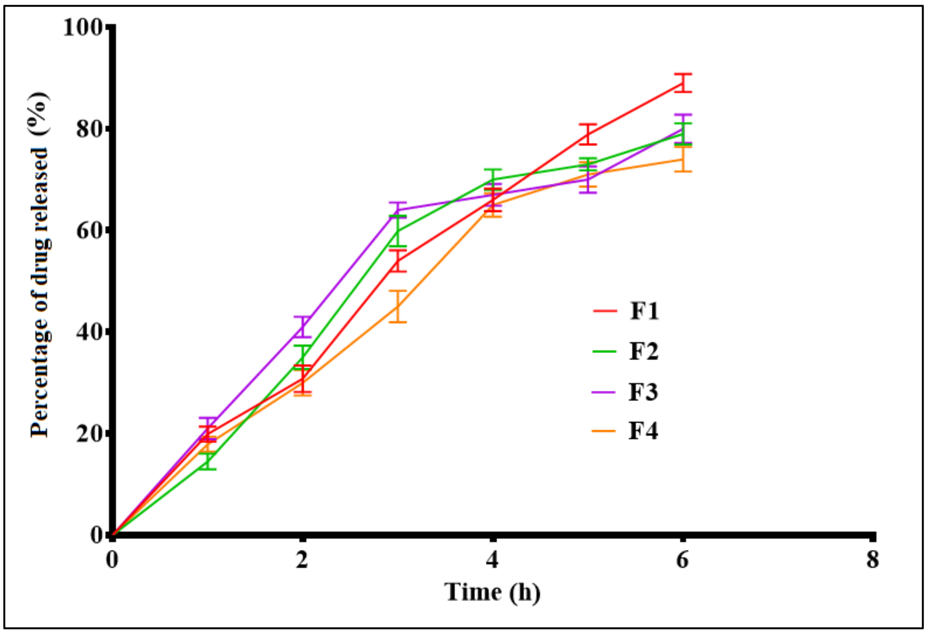

Figure S3. The release profiles of the formulated nanophytosomal dispersions

References

[1] Tas, Ç., Y. Özkan, A. Savaser, et al., 2003. In vitro release studies of chlorpheniramine maleate from gels prepared by different cellulose derivatives. Il Farmaco. 58, 605-611. https://doi.org/https://doi.org/10.1016/S0014-827X(03)00080-6

[2] Abdellatif, M. M., Y. E. Elakkad, A. A. Elwakeel, et al., 2021. Formulation and characterization of propolis and tea tree oil nanoemulsion loaded with clindamycin hydrochloride for wound healing: In-vitro and in-vivo wound healing assessment. Saudi Pharmaceutical Journal. 29, 1238-1249.

[3] Elakkad, Y. E., M. K. Younis, R. M. Allam, et al., 2021. Tenoxicam loaded hyalcubosomes for osteoarthritis. International journal of pharmaceutics. 601, 120483.

[4] Hou, Z., Y. Wang, C. Yin, et al., 2011. Terminal iterative learning control based station stop control of a train. International Journal of Control. 84, 1263-1274

[5] Soliman, S. M., N. A. Malak, O. N. El-Gazayerly, et al., 2010. Formulation of microemulsion gel systems for transdermal delivery of celecoxib: In vitro permeation, anti-inflammatory activity and skin irritation tests. Drug Discov Ther. 4, 459-471.

[6] Cartwright, A. C., 2016. The British pharmacopoeia, 1864 to 2014: medicines, international standards and the state, Routledge.

[7] Jiang, H., M. Fall, E. Yilmaz, et al., 2020. Effect of mineral admixtures on flow properties of fresh cemented paste backfill: Assessment of time dependency and thixotropy. Powder technology. 372, 258-266.
